# Analysis and Optimization of Rotationally Symmetric Au-Ag Alloy Nanoparticles for Refractive Index Sensing Properties Using T-Matrix Method

**DOI:** 10.3390/nano15131052

**Published:** 2025-07-06

**Authors:** Long Cheng, Shuhong Gong, Paerhatijiang Tuersun

**Affiliations:** 1 School of Physics, Xidian University, Xi’an 710071, China; chenglong3604@mail.ustc.edu.cn; 2 Xinjiang Key Laboratory for Luminescence Minerals and Optical Functional Materials, School of Physics and Electronic Engineering, Xinjiang Normal University, Urumqi 830054, China

**Keywords:** Au-Ag alloy nanoparticles, figure of merit, biosensing, T-matrix method, optimization

## Abstract

Previous investigations devoted to non-spherical nanoparticles for biosensing have primarily addressed two hot topics, namely, finding nanoparticles with the best shape for refractive index sensing properties and the optimization of size parameters. In this study, based on these hot topics, Au-Ag alloy nanoparticles with excellent optical properties were selected as the research object. Targeting rotationally symmetric Au-Ag alloy nanoparticles for biosensing applications, the complex media function correction model and T-matrix approach were used to systematically analyze the variation patterns of extinction properties, refractive index sensitivity, full width at half maximum, and figure of merit of three rotationally symmetric Au-Ag alloy nanoparticles with respect to the size of the particles and the Au molar fraction. In addition, we optimized the figure of merit to obtain the best size parameters and Au molar fractions for the three rotationally symmetric Au-Ag alloy nanoparticles. Finally, the range of dimensional parameters corresponding to a figure of merit greater than 98% of its maximum value was calculated. The results show that the optimized Au-Ag alloy nanorods exhibit a refractive index sensitivity of 395.2 nm/RIU, a figure of merit of 7.16, and a wide range of size parameters. Therefore, the optimized Au-Ag alloy nanorods can be used as high-performance biosensors. Furthermore, this study provides theoretical guidance for the application and preparation of rotationally symmetric Au-Ag alloy nanoparticles in biosensing.

## 1. Introduction

Metallic nanomaterials can efficiently interact with specific incident light (called resonant wavelengths), resulting in the occurrence of localized surface plasmon resonance (LSPR) phenomena [1,2]. Based on the LSPR properties of nanomaterials, they can strongly scatter and absorb incident light at resonance wavelengths and enhance and modulate of the local field strength of nanostructures [3,4,5,6]. Therefore, metallic nanomaterials have become a prominent research focus for bioimaging [7], surface-enhanced Raman scattering (SERS) [8], photothermal therapy [9,10], biosensing [11,12], and molecular detection [13].

In recent years, composite nanomaterials have attracted much attention as they can overcome the deficiencies of ordinary nanoparticles. These nanomaterials not only have the properties of their constituents but can also exhibit new properties due to the synergistic effect of the combination of different base materials [14,15,16,17]. Au nanoparticles exhibit excellent biocompatibility and chemical stability and demonstrate stable plasmonic responses [18,19]. Ag nanoparticles demonstrate outstanding refractive index sensing characteristics, a pronounced nonlinear optical response, and significant photocatalytic performance [20,21]. Au-Ag alloy nanoparticles, synthesized to combine the advantages of the two materials, have excellent optical properties, enhanced biocompatibility, improved structural stability, and excellent refractive index sensitivity [22,23]. Consequently, Au-Ag alloy nanoparticles demonstrate enhanced tissue penetration capacity during systemic delivery while exhibiting superior refractive index sensitivity to microenvironmental changes compared to their monometallic counterparts. Based on the above characteristics, Au-Ag alloy nanoparticles serve as highly effective biosensors for biotherapeutic applications [12,24,25].

In biosensing applications, refractive index sensitivity (RIS) and figure of merit (FOM) serve as the primary quantitative metrics for evaluating nanoparticle sensing performance [26]. The refractive index sensing properties of Au-Ag alloy nanoparticles depend on its LSPR properties, which can be modulated by varying the particle size, shape, material, and surrounding medium [4,19]. Therefore, the RIS and FOM of Au-Ag alloy nanoparticles for biosensing applications are highly dependent on the size and shape of the particles. In order to find metal nanoparticles with high RIS and an optimal FOM, researchers have investigated the refractive index sensing properties of several typical shapes of metal nanoparticles, such as nanospheres, nanorods, nanocubes, and nanodiscs. In 2017, Du et al. [27] performed a numerical analysis of the refractive index sensitivity of sensors for individual Ag nanoparticles, including nanodiscs, nanocubes, nanoprisms, and nanoparticle clusters. In 2024, Prajna et al. [28] systematically engineered Au nanoparticles with controlled morphologies (nanospheres: 20–50 nm; nanorods: aspect ratios 2–5) via seed-mediated growth, analyzing the refractive index sensitivity of the nanoparticles through combined UV-Vis spectroscopy and the FDTD method. Furthermore, researchers have examined the refractive index sensing properties of metal nanoparticles with complex shapes, including nanodonuts, nanostars, and core–shell structures, as well as their morphological characteristics. In 2023, Wang et al. [29] conducted a numerical investigation into the impact of eccentricity and splitting angle on the surface enhanced resonance spectroscopy (SERS) and refractive index sensing of TiN nanostars, employing the finite element method. In 2025, Santos et al. [30] synthesized nanodendrites and investigated the morphology of gold, silver, and gold–silver nanodendrite alloys; these nanodendrites were then deposited onto the surfaces of optical fibers, with the objective of enhancing the sensing properties of the fibers. In 2025, Yu et al. [31] discussed the application of gold–silver bimetallic nanoparticles in the surface-enhanced Raman scattering (SERS) detection of food contaminants in a review article. The article pointed out that both Au-Ag alloy nanoparticles and Au-Ag nanoparticles exhibit excellent plasmonic resonance characteristics and Raman enhancement effects. However, core–shell structures offer significant advantages in terms of electric field enhancement and resonance tuning, while alloy structures exhibit superior biocompatibility and stability. Despite the promising potential of nanoparticles with intricate structures and materials for achieving enhanced refractive index sensing properties, their restricted accessibility and challenges in preparation impede their suitability for large-scale production and clinical application. Therefore, it is necessary to find Au-Ag alloy nanoparticles with simple shapes and high refractive index sensing properties, as well as to investigate the optimal size parameters of Au-Ag alloy nanoparticles to maximize their refractive index sensing properties.

At the present time, there is a lack of comprehensive research on the geometric and compositional optimization of the refractive index sensing properties of rotationally symmetric Au-Ag alloy nanoparticles using semi-analytical electromagnetic modeling methods. The prevailing methodologies utilized for modeling the optical characteristics of metal nanoparticles encompass the following: Mie theory [32], T-matrix [33,34,35], discrete dipole approximation (DDA) [36], and finite-difference time-domain (FDTD) [37]. Mie theory is a rigorous analytical approach that is mainly employed to investigate the optical properties of spherical nanoparticles such as nanospheres and nanoshells. The T-matrix is a semi-analytical method that is mainly used to study the optical properties of rotationally symmetric nanoparticles with arbitrary orientation. DDA and FDTD are typical numerical methods that enable rigorous analysis of optical responses in nanoparticles with arbitrary geometries. Unlike analytical approaches such as Mie theory and the T-matrix method, DDA and FDTD face a critical precision/efficiency trade-off: refining their discretization parameters (dipole density in DDA, grid resolution in FDTD) improves accuracy but causes the computational time to increase exponentially, especially when modeling nanoparticles with complex geometries. Considering the abovementioned characteristics, we decided to use the T-matrix approach in this research.

In this study, the refractive index sensing characteristics of Au-Ag alloy nanospheroids, nanocylinders, and nanorods are theoretically and systematically analyzed and optimized using the T-matrix method and the size correction model of the alloy dielectric function. The optimal quality factors and the corresponding size parameters and Au mole fraction ranges for three shapes are obtained. The results of this study provide theoretical guidance and data support for the synthesis and shape control of gold–silver alloy nanoparticles and their application in biosensing.

## 2. Materials and Methods

**Figure 1:**
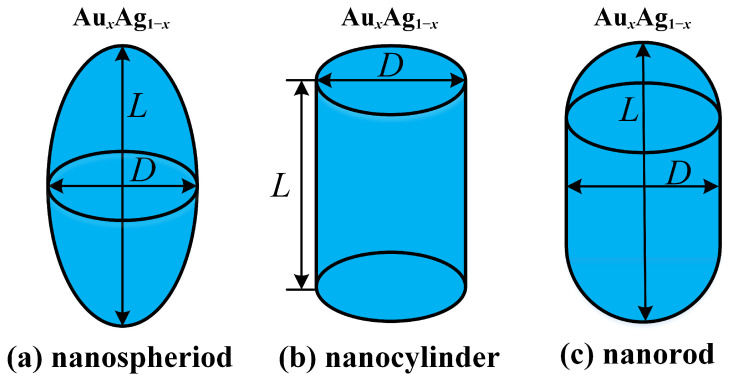
Geometric modeling of rotationally symmetric Au-Ag alloys: (**a**) nanospheroid, (**b**) nanocylinder, and (**c**) nanorod.

This study investigated Au-Ag alloy nanoparticles with rotational symmetry, specifically nanospheroids, nanocylinders, and nanorods, whose geometric configurations are illustrated in Figure 1. The dimensional parameters of these three types of rotationally symmetric Au-Ag alloy nanoparticles are described by the length *L* and diameter *D*. The Au-Ag alloy nanorod is geometrically characterized by a cylindrical core (length *L*, diameter *D*) that is symmetrically capped with hemispherical ends (diameter *D*), as illustrated in Figure 1c. In addition to these two geometrical parameters, the size parameters of the particles can be described by the aspect ratio *R* = *L*/*D*. In particular, this study optimizes nanoparticle designs through two key parameters, *L* and *R*, focusing on prolate spheroidal and cylindrical geometries where axial dimensions dominate (*L* > *D*). The molar fractions of Au and Ag in the alloy are denoted as *x* and 1−*x*, respectively.

**Figure 2:**
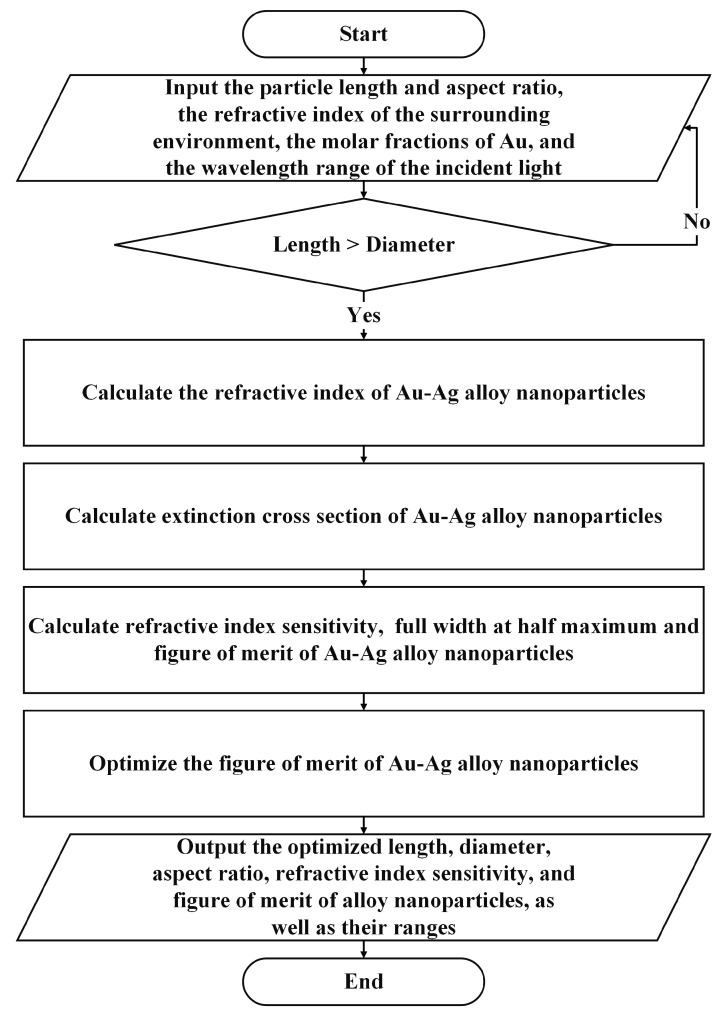
Schematic diagram of the analysis and optimization simulation workflow of the refractive index sensing characteristics of Au-Ag alloy nanoparticles.

Figure 2 shows the modeling workflow from geometric input to optical output. First, we determine the size relationship between *L* and *D*. If *L* > *D*, we perform size correction on the dielectric function of the Au-Ag alloy nanoparticles. Otherwise, we return to the input step. Second, we use the T-matrix method to calculate the extinction characteristics, refractive index sensitivity (RIS), full width at half maximum (FWHM), and figure of merit (FOM) of randomly oriented Au-Ag alloy nanoparticles. Then, we optimize the FOM of the gold–silver alloy nanoparticles. Finally, we output the optimized size parameters and refractive index sensing characteristic parameters of the Au-Ag alloy nanoparticles, along with their respective ranges.

In addition, the orientation distribution of the nanoparticles is random, and the incident light is irradiated in an arbitrary direction in practical studies. Therefore, the T-matrix method is used in this study to model the extinction properties of randomly oriented rotationally symmetric Au-Ag alloy nanoparticles. This method employs the random orientation model of nanoparticles reported in the paper by Mishchenko et al. [38]. When studying randomly oriented non-spherical rotationally symmetric nanoparticles, the directional averaging caused by random orientation eliminates polarization dependence, resulting in extinction characteristics that are determined solely by factors such as particle shape, size, material, surrounding medium, and wavelength. The extinction properties are obtained from the absorption and scattering properties, which can be described by the extinction cross-section and is calculated for uniform and randomly oriented rotationally symmetric particles as follows [39]:

(1) $$\langle C_{\mathrm{abs}}\rangle =-\frac{2\pi }{k^{2}}Re\sum_{n=1}^{\infty }\sum_{m=-n}^{n}\left[T_{mnmn}^{11}+T_{mnmn}^{22}\right]-\frac{2\pi }{k^{2}}\sum_{m=1}^{\infty }\sum_{m=-n}^{n}\sum_{n^{\prime }=1}^{\infty }\sum_{m^{\prime }=-n^{\prime }}^{n^{\prime }}\sum_{k=1}^{2}\sum_{l=1}^{2}{\left|T_{mnm^{\prime }n^{\prime }}^{kl}\right|}^{2},$$

(2) $$\langle C_{\mathrm{sca}}\rangle =\frac{2\pi }{k^{2}}\sum_{m=1}^{\infty }\sum_{m=-n}^{n}\sum_{n^{\prime }=1}^{\infty }\sum_{m^{\prime }=-n^{\prime }}^{n^{\prime }}\sum_{k=1}^{2}\sum_{l=1}^{2}{\left|T_{mnm^{\prime }n^{\prime }}^{kl}\right|}^{2},$$

(3) $$\langle C_{\mathrm{ext}}\rangle =\langle C_{\mathrm{abs}}\rangle +\langle C_{\mathrm{sca}}\rangle =-\frac{2\pi }{k^{2}}Re\sum_{n=1}^{\infty }\sum_{m=-n}^{n}\left[T_{mnmn}^{11}+T_{mnmn}^{22}\right],$$

where *C*_abs_, *C*_sca_, and *C*_ext_ denote the average absorption cross-section, the average scattering cross-section, and the average extinction cross-section of rotationally symmetric particles. *k* is the wavelength of the incident light in the surrounding medium, and $T_{mnmn}^{11}$ and $T_{mnmn}^{22}$ are the diagonal elements of the T-matrix. The T-matrix calculation is performed in the particle’s coordinate system, and the results are independent of the incident wave direction. When calculating the average response, all possible orientations are automatically and completely taken into account through the analytical rotation and averaging process. After the above precise analysis, the orientation-averaged optical cross-section (such as extinction spectrum) no longer exhibits the anisotropic characteristics inherent to individual particles. The T-matrix is a transformation matrix that represents the relationship between the scattered field expansion coefficients and the incident field expansion coefficients, and the column vectors of the scattered field expansion coefficients are obtained by multiplying the T-matrix with the column vectors of the incident field expansion coefficients. The formula shows that the mean value of the extinction cross-section for each particle over a uniformly oriented distribution is proportional to the real part of the sum of the diagonal elements of the T-matrix computed in the particle’s frame of reference.

For randomly oriented non-spherical nanoparticle systems, the orientation of nanoparticles is uniformly distributed in all spatial directions. At this point, the local surface plasmon resonance response of the system is formed by the superposition of the responses of all possible oriented particles. We used the T-matrix method to calculate the extinction cross-section of the randomly oriented system and extracted the resonance wavelength *λ_res_* and full width at half maximum (FWHM). The refractive index sensitivity (RIS) is defined as the rate of change in the position of the localized surface plasmon resonance (LSPR) peak *λ_res_* in the system’s extinction spectrum with respect to the refractive index ∆*n_m_* of the surrounding medium [40]:

(4) $$RIS=\frac{\Delta \lambda_{res}}{\Delta n_{m}},$$

where ∆*n_m_* represents the change in the refractive index of the medium and ∆*λ_res_* represents the shift in the resonance wavelength. The unit for refractive index sensitivity (RIS) is nm/RIU, where RIU is the English abbreviation for refractive index unit. Notably, the primary research environment targeted in this study is human subcutaneous adipose tissue, which has a refractive index of 1.44 [41,42]. Consequently, the refractive indices of the surrounding medium selected in this paper are 1.35, 1.45, 1.55, and 1.65.

Defining the figure of merit can help to better characterize the biosensing properties of Au-Ag alloy nanoparticles; the figure of merit (FOM) is defined as the ratio of the sensitivity of the resonance peak to the full width at half maximum (FWHM):

(5) $$FOM=\frac{RIS}{FWHM},$$

Due to anisotropy, the sensitivity of a single particle depends on the angle between the incident electric field and the particle’s principal axis. Under random orientation, the average sensitivity of the system lies between the sensitivity in the parallel direction (*S*_⊥_) and the sensitivity in the perpendicular direction (*S*_||_). Additionally, the non-uniform broadening of the resonance peak results in the system’s FWHM being greater than that of a single-oriented particle [43]. Therefore, the quality factor of a randomly oriented system is typically lower than that of a single-oriented particle in its optimal direction (i.e., the direction of maximum sensitivity).

For accurate calculation of the extinction properties in Au-Ag alloy nanoparticles, the particle refractive index must be properly accounted for, which is determined as follows:

(6) $$n_{p}=\sqrt{\varepsilon (\omega ,x)},$$

where *n_p_* is the refractive index of the particles and *ε*(*ω*,*x*) is the dielectric constant of alloys.

When light interacts with metal nanoparticles, particularly those smaller than the mean free path of free electrons, the refractive index of the nanoparticles deviates from the actual value. This deviation is attributed to the collisions between free electrons and the nanoparticle surface, which become a significant factor and cannot be disregarded. Consequently, the refractive index of nanoparticles is intricately linked to their size. In this investigation, we employ the alloy dielectric function model established by Rioux et al. [44], which encompasses the Drude term and two critical points. This model accounts for the optical frequency, alloy composition, and damping coefficient and performs size-dependent correction of the damping coefficient. Furthermore, the model shows excellent agreement with experimental results. The complex permittivity of the alloy nanoparticles can be expressed as

(7) $$\begin{array}{cc} \varepsilon (\omega ,x) & =\varepsilon_{\infty }-\frac{{\left(\omega_{p}\left(x\right)\right)}^{2}}{\omega^{2}+i\omega \Gamma_{p}\left(x\right)}\\ & +\varepsilon_{cp1}\left(\omega ,\omega_{01}\left(x\right),\omega_{g1}\left(x\right),\Gamma_{1}\left(x\right),A_{1}\left(x\right)\right)\\ & +\varepsilon_{cp2}\left(\omega ,\omega_{02}\left(x\right),\omega_{g1}\left(x\right),\Gamma_{2}\left(x\right),A_{2}\left(x\right)\right) \end{array},$$

where *ω* represents the angular frequency of the incident light, *x* denotes the mole fraction of Au, *ε*_∞_ signifies the high-frequency dielectric constant originating from interband electronic transitions, *ω_p_* stands for the plasma frequency of free electrons, Γ*_p_* indicates the damping coefficient in the Drude term (the broadening factor related to the collision frequency of free electrons), *ε_cp_*_1_ and *ε_cp_*_2_ represent the interband dielectric contributions at two critical points, *ω*_01_ and *ω*_02_ denote the characteristic transition frequencies at two critical points, *ω_g_*_1_ signifies the energy band gap at the critical point, Γ_1_ and Γ_2_ are the collision frequency of free electrons at two critical points, and *A*_1_ and *A*_2_ denote the amplitude parameters at two critical points [23].

Moreover, the electron damping coefficient Γ_p_ in nanoparticles is primarily determined by the electron mean free path. In Au-Ag alloy nanoparticles, increased scattering due to chemical disorder decreases the effective mean free path, resulting in heightened damping in comparison to pure metals. The expression for Γ_p_ is as follows [45]:

(8) $$\Gamma_{p}(L_{\mathrm{eff}})=\Gamma_{\mathrm{pBulk}}+\alpha \frac{h\nu_{f}}{L_{\mathrm{eff}}},$$

where *Γ*_pBulk_ is the damping coefficient of the bulk material, determined through parameter fitting; *α* is a dimensionless parameter, which is usually considered to be close to 1 (taken as 1 in the text); *h* is the Planck constant; *ν_f_* represents the Fermi velocity of the free electrons, equal to 1.4 × 10^6^ m/s for Au-Ag alloys [44]; and *L*_eff_ denotes the effective mean free path of free electrons within the nanoparticles [46].

Many parameters in the dielectric function formalism (Equation (7)) for alloy nanoparticles are dependent on the Au mole fraction *x*. The plasma frequency for a Au-Ag alloy with a variable x can be expressed as

(9) $$\omega_{p}\left(x\right)=x^{2}\left(2\omega_{pAu}-4\omega_{pAu0.5Ag0.5}+2\omega_{pAg}\right)+x\left(-\omega_{pAu}+4\omega_{pAu0.5Ag0.5}-3\omega_{pAg}\right)+\omega_{pAg},$$

where *ω_pAu_*, *ω_pAg_*, and *ω_pAu0.5Ag0.5_* correspond to the plasma frequencies of monometallic Au, monometallic Ag, and an equimolar (50%–50%) Au-Ag alloy system, respectively [23]. This pattern of composition dependence applies to all other model parameters as well. In the dielectric function model described by Rioux et al. (Equations (7)–(9)), a genetic algorithm was employed to optimize the fitting of multiple experimental datasets, thereby determining all undetermined parameters within the model, as shown in Appendix A of Table A1.

For the theoretical calculations, Fortran code was used for calculating the extinction cross-section, the refractive index sensitivity, and the figure of merit for three rotationally symmetric Au-Ag alloy nanoparticles with uniform and random orientations. The code was developed based on the open-source T-matrix program written by Mishchenko et al. https://www.giss.nasa.gov/staff/mmishchenko/tmatrix (accessed on 4 July 2025) [34]. Additionally, a size-corrected model for the dielectric function of Au-Ag alloy nanoparticles was integrated into the code. In the calculations in this paper, we considered Au-Ag alloy nanoparticles with lengths ranging from 20 to 100 nm, aspect ratios between 2 and 6, and Au mole fractions varying from 0 to 1. The step sizes for length, aspect ratio, and Au mole fraction were set at 1 nm, 0.1, and 0.1, respectively.

## 3. Results and Discussion

**Figure 3:**
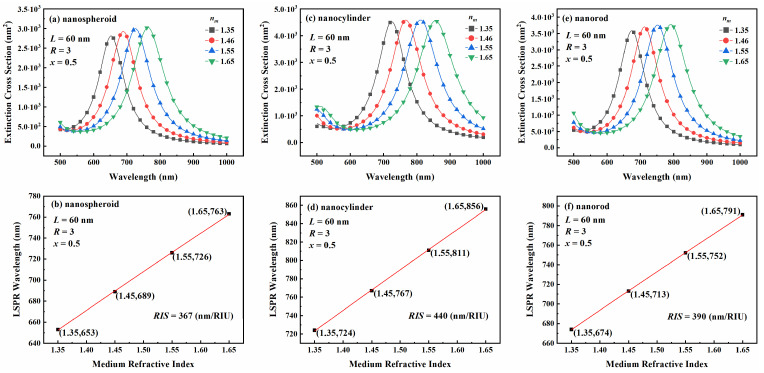
Extinction spectra and LSPR wavelength variation with refractive index of surrounding medium for Au-Ag alloys: (**a**,**b**) nanospheroid, (**c**,**d**) nanocylinder, and (**e**,**f**) nanorod. Refractive index sensitivity (RIS) for all Au-Ag alloy nanoparticles.

The refractive index sensing capabilities of rotationally symmetric Au-Ag alloy nanoparticles depend on their extinction spectral characteristics. As shown in Figure 3, the influence of the surrounding medium’s refractive index on the extinction spectra and LSPR wavelengths was analyzed for the three nanoparticle shapes. The nanoparticles were configured with a length of 60 nm, an aspect ratio of 3, and a Au molar fraction of 0.5. Furthermore, a nonlinear regression analysis was conducted on the results at the LSPR wavelength. As can be seen in Figure 3, when the refractive index of the surrounding medium increases from 1.35 to 1.65, the extinction capacity of the Au-Ag alloy nanospheroid gradually increases and its extinction resonance wavelength redshifts from 653 nm to 862 nm, corresponding to a refractive index sensitivity of 367 nm/RIU (Figure 3a,b). In contrast, the extinction capacity of the nanocylinder remains nearly constant, but its resonance wavelength redshifts from 724 nm to 856 nm, corresponding to a refractive index sensitivity of 440 nm/RIU (Figure 3c,d). For the nanorod, the extinction capacity increases gradually, and the resonance wavelength redshifts from 675 nm to 791 nm, corresponding to a refractive index sensitivity of 390 nm/RIU (Figure 3e,f). The observed redshift in the LSPR wavelengths of all three nanoparticle types is attributed to the increase in the refractive index of the surrounding medium. This phenomenon occurs due to the reduction in the coulomb force between electrons and nuclei within the nanoparticles as the refractive index increases. Consequently, the electron–wall collision frequency decreases, leading to lower resonance frequencies and longer resonant wavelengths, ultimately resulting in the redshift phenomenon.

**Figure 4:**
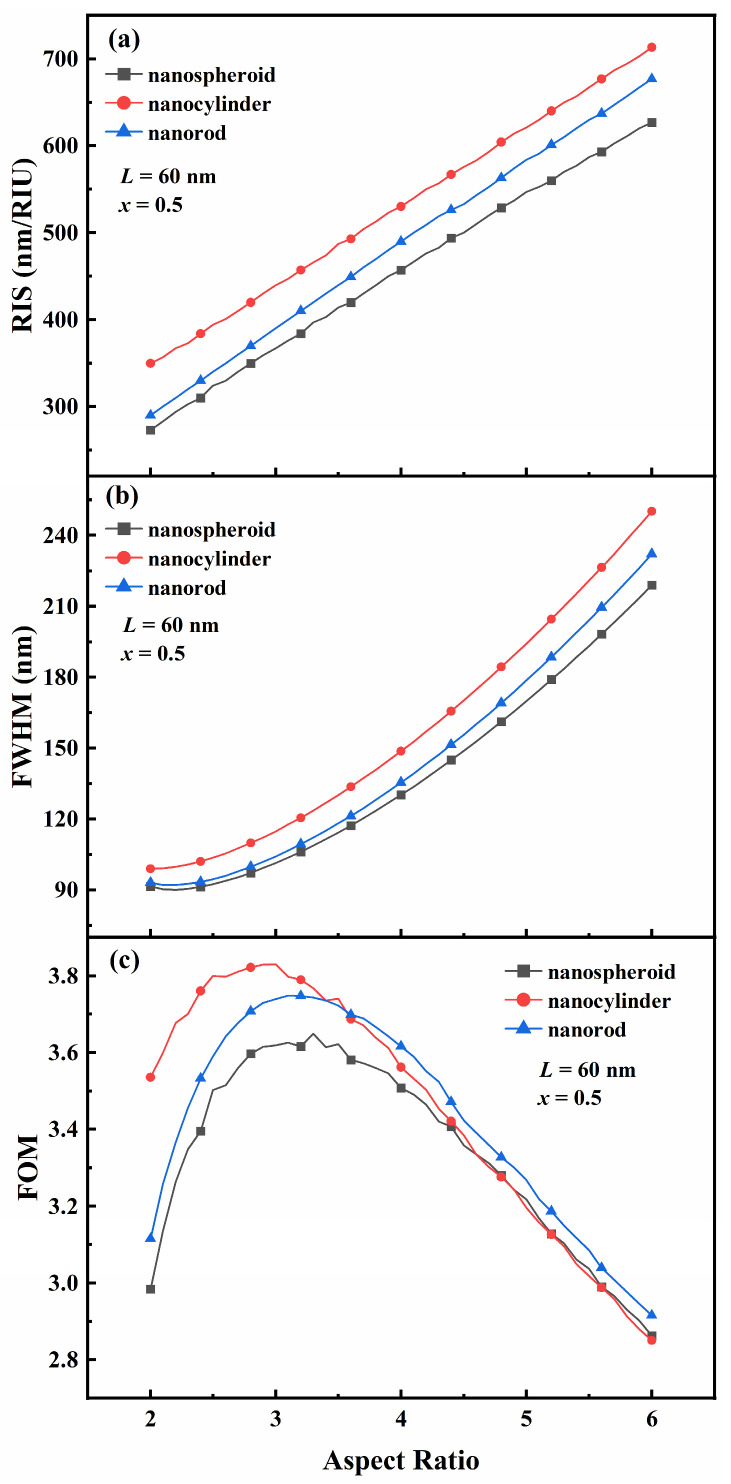
Effect of the aspect ratio *R* of Au-Ag alloy nanospheroids, nanocylinders, and nanorods on (**a**) refractive index sensitivity (RIS), (**b**) full width at half maximum (FWHM), and (**c**) figure of merit (FOM).

In order to study the effect of the aspect ratio (*R*) of the particles on the refractive index sensitivity (RIS), full width at half maximum (FWHM), and figure of merit (FOM) of Au-Ag alloy nanospheroids, nanocylinders, and nanorods, the aspect ratio was altered from 2 to 6 with a step size of 0.1; the other properties were a Au mole fraction (*x*) of 0.5 and a fixed length of 60 nm (Figure 4). Due to the increase in aspect ratio, the RIS of the three Au-Ag alloy nanoparticles increases almost linearly (Figure 4a), the FWHM of the three Au-Ag alloy nanoparticles increases almost exponentially (Figure 4b), and the FOM of the three Au-Ag alloy nanoparticles first increases and then decreases (Figure 4c). On the surface, when length remains constant, as *R* increases, the growth rate of the FWHM is faster than that of RIS, which is the reason for the change in the FOM of Au-Ag alloy nanoparticles. However, the sensitivity enhancement caused by the resonance redshift and the weakening of radiation damping are the main intrinsic factors leading to the change in FOM. Among them, the sharp decrease in radiation damping causes spectral broadening, resulting in a significant increase in FWHM as *R* increases (Equations (4)–(9)). As can be seen from the Figure 4, the Au-Ag alloy nanocylinders exhibit the largest refractive index sensitivity and full width at half maximum width at all aspect ratios. The maximum FOM of 3.65 for the Au-Ag alloy nanospheroids corresponds to an aspect ratio of 3.3, the maximum FOM of 3.83 for the Au-Ag alloy nanocylinder corresponds to an aspect ratio of 3.0, and the maximum FOM of 3.75 for the Au-Ag alloy nanorod corresponds to an aspect ratio of 3.1. Moreover, the Au-Ag alloy nanocylinders have the largest FOM among the three Au-Ag alloy nanoparticles when the aspect ratio is in the range of 0–3.6. As the aspect ratio increases from 3.6 to 6.0, the FOM of Au-Ag alloy nanocylinders decreases significantly, eventually falling below that of Au-Ag alloy nanospheroids. Notably, Au-Ag alloy nanorods exhibit a favorable FOM among these structures.

**Figure 5:**
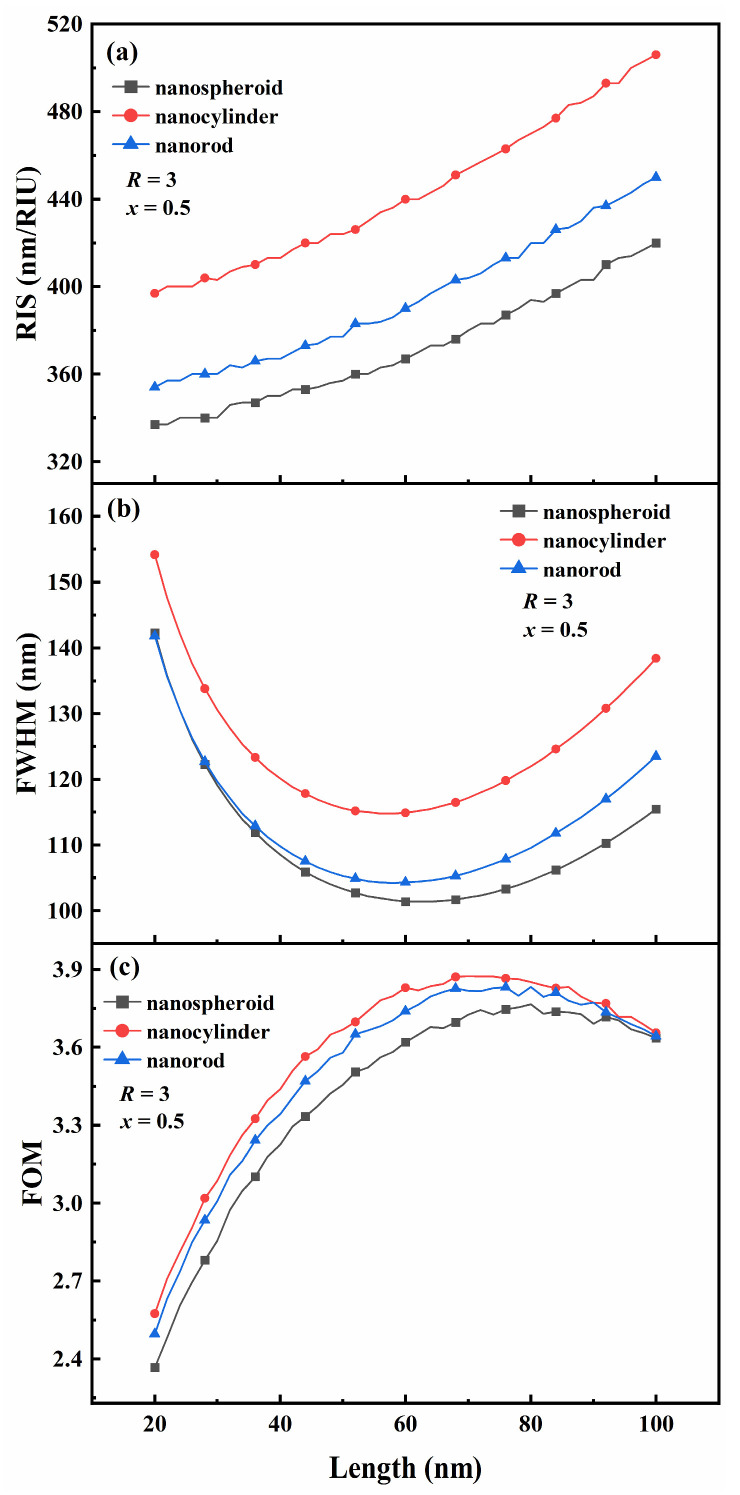
Effect of the length *L* of Au-Ag alloy nanospheroids, nanocylinders, and nanorods on (**a**) refractive index sensitivity (RIS), (**b**) full width at half maximum (FWHM), and (**c**) figure of merit (FOM).

Figure 5 shows the effect of the length (*L*) of the three Au-Ag alloy nanoparticles on the RIS, FWHM, and FOM. For this experiment, the length was chosen to be between 20 and 100 nm, with a step size of 1; the Au molar fraction was 0.5; and the aspect ratio was fixed at 3. From the Figure 5, when R remains constant, as the length is increased, the resonance wavelength of Au-Ag alloy nanoparticles shifts toward red light, the radiation damping increases and then decreases, and the RIS of the three Au-Ag alloy nanoparticles increases almost linearly (Figure 5a). Additionally, the FWHM of the three Au-Ag alloy nanoparticles decreases and then increases and has a minimum value within the length interval of 50–70 nm (Figure 5b). The FOM of the three Au-Ag alloy nanoparticles exhibits a trend of an initial increase followed by a gradual decrease, and the maximum value for the FOM occurs in the length interval of 60–80 nm (Figure 5c). The maximum FOM of the Au-Ag alloy nanospheroids is 3.77, corresponding to a length of 80 nm. The maximum FOM of the Au-Ag alloy nanocylinder is 3.87, corresponding to a length of 70 nm. The maximum FOM of the Au-Ag alloy nanorods is 3.83, corresponding to a length of 80 nm. The Au-Ag alloy nanocylinders exhibited the maximum RIS, FWHM, and FOM at all lengths.

**Figure 6:**
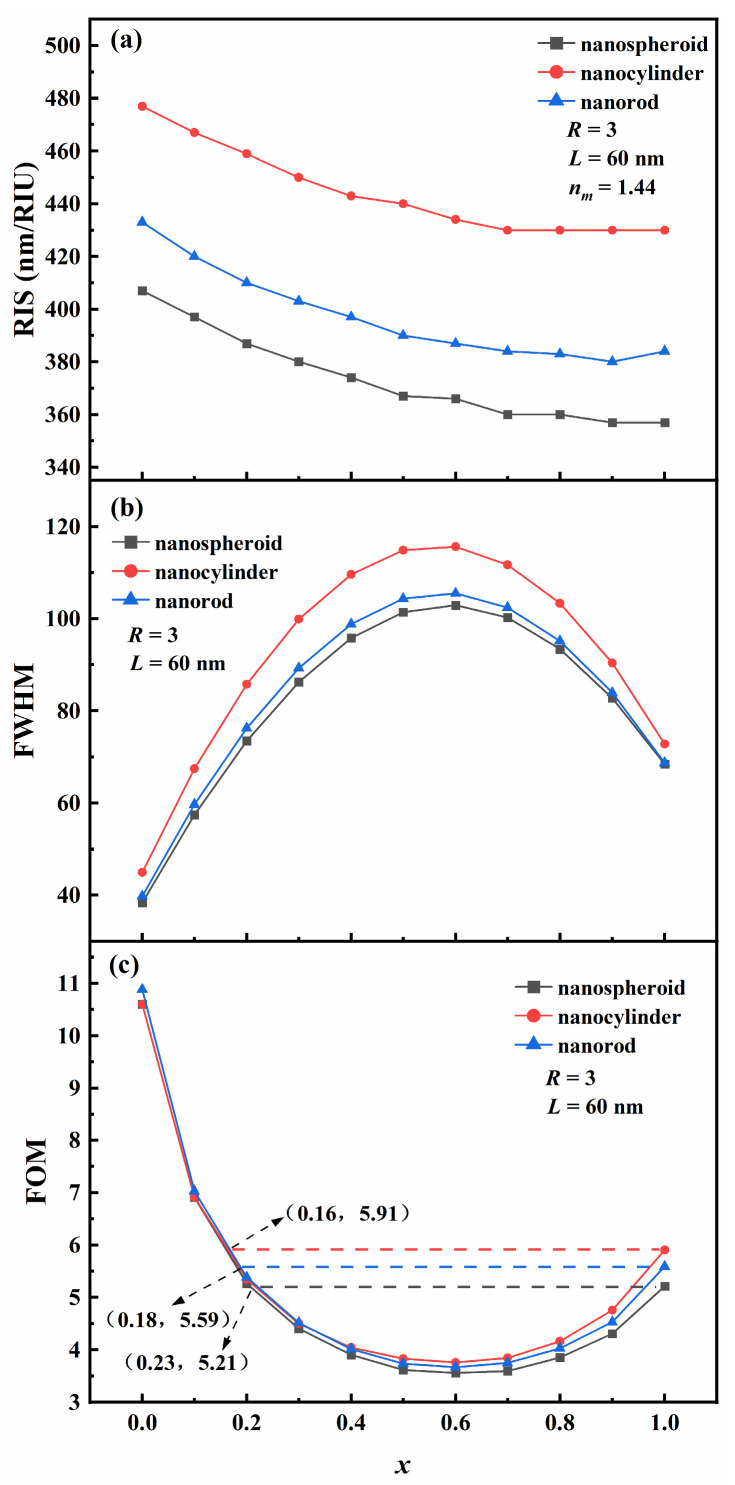
Effect of the Au molar fraction *x* of Au-Ag alloy nanospheroids, nanocylinders, and nanorods on (**a**) refractive index sensitivity (RIS), (**b**) full width at half maximum (FWHM), and (**c**) figure of merit (FOM).

The Au molar fraction (*x*) is also an important parameter affecting the refractive index sensing properties of Au-Ag alloy nanoparticles. We systematically analyzed the effect of the *x* on RIS, FWHM, and FOM for the three Au-Ag alloy nanoparticles, as shown in Figure 6. The *x* was chosen to be between 0 and 1, the step size was 0.1, the length was 60 nm, and the aspect ratio was fixed at 3. When the *x* is increased, the electron density per unit volume of the nanoparticles gradually decreases, leading to a decrease in the plasma frequency, which in turn causes the resonance wavelength to shift toward the blue end of the spectrum and the radiation damping to first decrease and then increase. This further contributes to the RIS of the three Au-Ag alloy nanoparticles decreasing and then increasing; the RIS has a minimum value within the *x* interval of 0.2–0.4 (Figure 6a). The FWHM of the three Au-Ag alloy nanoparticles exhibits a trend of initial increase followed by a gradual decrease and has a maximum value at *x* = 0.6 (Figure 6b). The FOM of the three Au-Ag alloy nanoparticles decreases and then increases and has a minimum value at *x* = 0.6 (Figure 6c). Clearly, all three particles correspond to the maximum FOM when the *x* is zero. The maximum FOM values are 10.59, 10.6, and 10.88 for Au-Ag alloy nanospheroids, nanocylinders, and nanorods, respectively. In addition, the *x* should be chosen to be between 0 and 0.23, 0 and 0.16, and 0 and 0.18 when calculating the FOM of the Au-Ag alloy nanospheroids, nanocylinders, and Au-Ag alloy nanorods, respectively, (*x* ≠ 0 for all cases) because this not only avoids the toxicity and oxidizing properties of pure silver nanoparticles and the disadvantages of pure gold nanoparticles, which are expensive and not easily available, but also ensures that the alloy nanoparticles have good refractive index sensing properties.

**Figure 7:**
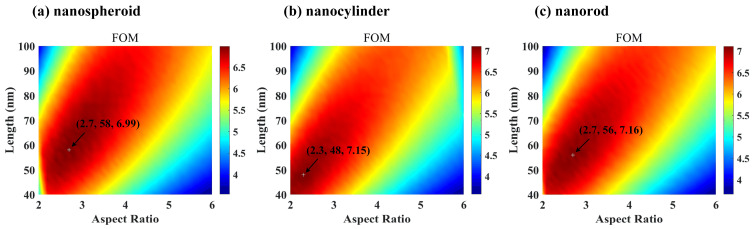
Variation in the figure of merit (FOM) of Au-Ag alloy (**a**) nanospheroids, (**b**) nanocylinders, and (**c**) nanorods as a function of aspect ratio and length. The Au molar fraction is 0.1. The plus (+) sign indicates the point of maximum FOM, and the numbers in parentheses represent the optimal aspect ratio, optimal length, and maximum FOM, respectively.

In order to study the sensing characteristics of the three Au-Ag alloy nanoparticles more deeply and determine the optimal size parameters, we calculated the figure of merit (FOM) values for these nanoparticles at a Au mole fraction *x* of 0.1 while varying the aspect ratio and length, as depicted in Figure 7. Clearly, each of the three Au-Ag alloy nanoparticles exhibited a distinct peak representing the maximum FOM. The data obtained from this investigation suggest that optimized Au-Ag alloy nanorods, with an aspect ratio of 2.7 and a length of 56 nm, demonstrated superior refractive index sensing capabilities compared to both optimized Au-Ag alloy nanospheroids and nanocylinders. Specifically, the maximum FOM of the Au-Ag alloy nanorods surpassed that of the Au-Ag alloy nanospheroids by 2.4% and marginally exceeded that of the Au-Ag alloy nanocylinders.

**Figure 8:**
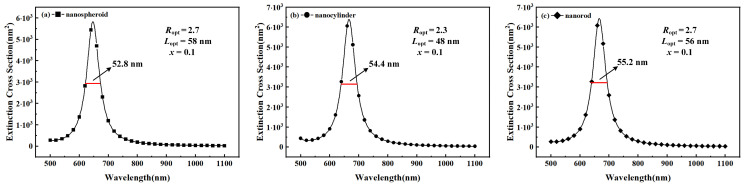
Extinction spectra and half-peak widths of the Au-Ag alloy (**a**) nanospheroids, (**b**) nanocylinders, and (**c**) nanorods after optimization. The Au molar fraction is 0.1. The horizontal line indicates the width of the full width at half maximum (FWHM).

To obtain the optimized extinction spectra of the three Au-Ag alloy nanoparticles, their extinction cross-sections under the optimum size parameter conditions were calculated at a Au molar fraction *x* of 0.1, as shown in Figure 8. The figure shows that the resonance wavelengths and FWHM values of Au-Ag alloy nanospheroids, nanocylinders, and nanorods are 647 nm and 52.8 nm, 666 nm and 54.4 nm, and 667 nm and 55.2 nm, respectively.

In order to provide more comprehensive data, the optimization results for the three Au-Ag alloy nanoparticles at a Au molar fraction *x* of 0.1 are given in Table 1. Obviously, Au-Ag alloy nanocylinders have the best optimization results in terms of refractive index sensitivity (RIS_opt_) and figure of merit (FOM_opt_). The refractive index sensitivities corresponding to the optimal quality factors for the three Au-Ag alloy nanoparticles were 369.1 nm/RIU, 387.0 nm/RIU, and 395.0 nm/RIU, respectively. The optimal refractive index sensitivity of the Au-Ag alloy nanocylinders was 7.1% higher than that of the Au-Ag alloy nanospheroids and 2.1% higher than that of the Au-Ag alloy nanocylinders. This implies that Au-Ag alloy nanocylinders have good potential for biosensing applications.

**Figure 9:**
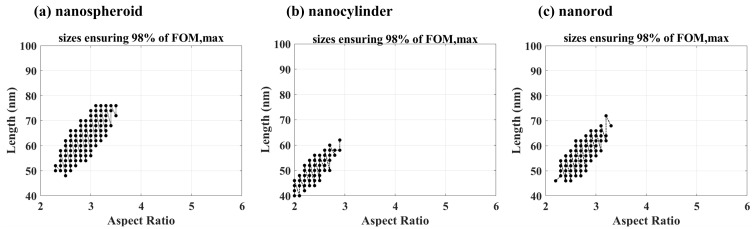
Aspect ratio and length of the Au-Ag alloy (**a**) nanospheroids, (**b**) nanocylinders, and (**c**) nanorods, ensuring that the figure of merit (FOM) values are larger than 98% of their maximum values. The Au molar fraction is 0.1.

The above analysis shows that there is only one set of optimal size parameters for a particular biological tissue and Au molar fraction. However, it is difficult to prepare Au-Ag alloy nanoparticles at the optimal size. Therefore, it is crucial to calculate the range of dimensional parameters for which the figure of merit is greater than one of its thresholds. In this study, we assumed the threshold to be 98% of the best figure of merit (FOM) value. Figure 9 represents the size parameters of the three shapes of Au-Ag alloy nanoparticles that achieve 98% of the maximum value of their figure of merit for a Au molar fraction of 0.1 and an increase in the surrounding refractive index from 1.35 to 1.65 in steps of 0.1. From Figure 9, it can be seen that among the three shapes of Au-Ag alloy nanoparticles, the size range of nanospheroids is larger that of nanocylinders is beyond the boundary. This means that Au-Ag alloy nanocylinders have a smaller particle size than Au-Ag alloy nanorods for the same quality factor. However, the above size parameters alone are not sufficient to provide data support for the preparation of Au-Ag alloy nanoparticles in three shapes.

In order to give more detailed dimensional parameters for the three shapes of Au-Ag alloy nanoparticles, we calculated the minimum, maximum, mean, and standard deviation of the aspect ratios and lengths of the three Au-Ag alloy nanoparticles that satisfy the 98% optimal figure of merit threshold at a Au molar fraction of 0.1 in a biological tissue environment, as shown in Table 2.

## 4. Conclusions

In this paper, for the application of Au-Ag alloy nanoparticles in biosensing, the refractive index sensing properties of Au-Ag alloy nanospheroids, nanocylinders, and nanorods were analyzed and optimized by using Au-Ag alloy size-corrected modeling and T-matrix methods. In this study, first, the effects of aspect ratio (*R*), length (*L*), and Au mole fraction (*x*) on the refractive index sensitivity (RIS), full width at half maximum (FWHM), and figure of merit (FOM) of three shapes of Au-Ag alloy nanoparticles were systematically analyzed. Secondly, the optimal figure of merit and corresponding optimal size parameters were obtained through optimization. Finally, the size parameters of three shapes of Au-Ag alloy nanoparticles satisfying the greater than 98% of the maximum value of their figure of merit threshold were calculated and presented. The results show that the extinction properties of the three shapes of Au-Ag alloy nanoparticles are very sensitive to changes in the refractive index of the surroundings. The effect of the Au molar fraction *x* on the quality factor is more pronounced than the size parameter of the particles. In order to avoid the drawbacks of toxicity and oxidizability associated with pure Ag and the expensive and not readily available pure Au, *x* takes the value (0–0.2 ± 0.03] as the optimal Au molar fraction interval for the preparation of the three shapes of Au-Ag alloy nanoparticles. The optimized Au-Ag alloy nanorods have a figure of merit and refractive index sensitivity of 7.16 and 395.2 nm/RIU, respectively, their refractive index sensing characteristics are better than those of Au-Ag alloy nanospheroids and nanocylinders, and they have larger size parameters as well as a wider range. In addition, we have calculated and given detailed values for the aspect ratio and length for the three shapes of Au-Ag alloy nanoparticles to satisfy the figure of merit greater than 98% of its maximum value at threshold at a Au molar fraction of 0.1 in a biological tissue environment. This study investigated how the refractive index sensing properties of three shapes of Au-Ag alloy nanoparticles vary with size parameter and Au mole fraction in biological tissues. The optimal size parameter and Au mole fraction ranges were provided, providing data support and theoretical guidance for the application and preparation of these shapes of Au-Ag alloy nanoparticles in the field of biological sensing. Further studies on the refractive index sensing properties of nanoparticles using different shapes and materials, as well as on the mechanism by which the molar fraction of the alloy and Lorentz friction in surface plasmons influences the refractive index sensing properties will be subsequently carried out.

## Figures and Tables

**Table 1:** Optimization results of refractive index sensitivity RIS_opt_, quality factor FOM_opt_, Au mole fraction *x*_opt_, aspect ratio *R*_opt_, length *L*_opt_, resonance wavelength λ_opt_, and full width at half maximum FWHM_opt_ of the Au-Ag alloy nanospheroids, nanocylinders, and nanorods.

Parameters	Nanospheroid	Nanocylinder	Nanorod
*R*_opt_	2.7	2.3	2.7
*L*_opt_ (nm)	58	48	56
*x*_opt_	0.1	0.1	0.1
λ_opt_ (nm)	647	666	667
FOM_opt_	6.99	7.15	7.16
FWHM_opt_ (nm)	52.8	54.4	55.2
RIS_opt_ (nm/RIU)	369.1	387.0	395.2

**Table 2:** Minimum, maximum, mean, and standard deviation of aspect ratios *R* and lengths *L* of the Au-Ag alloy nanospheroids, nanocylinders, and nanorods with a figure of merit FOM greater than 98% of their maximum values. The Au molar fraction is 0.1.

Shape	[*R*_min_, *R*_max_]	*R*_mean_ ± σ_R_	[*L*_min_, *L*_max_] (nm)	*L*_mean_ ± σ_R_ (nm)
Nanospheroid	[2.3, 3.5]	2.9 ± 0.3	[48, 76]	63 ± 8
Nanocylinder	[2.0, 2.9]	2.4 ± 0.2	[40, 62]	50 ± 6
Nanorod	[2.2, 3.3]	2.7 ± 0.3	[46, 72]	57 ± 6

## Data Availability

Data are contained within the article. The data that support the findings of this study are available from the corresponding authors upon request.

## Appendix A

The fitting parameters of the Au-Ag alloy nanoparticle dielectric function model [44] used in this study are shown in Table A1.

**Table A1:** Fitted parameters of the dielectric model of Au-Ag alloy.

Metal	Ag	Au0.5Ag0.5	Au
*ω*_p_/eV	8.5546	9.0218	8.9234
Γ_p_/eV	0.022427	0.16713	0.042389
*ε*_∞_	1.7381	2.2838	2.2715
*ω*_g1_/eV	4.0575	3.0209	2.6652
*ω*_01_/eV	3.9260	2.7976	2.3957
Γ_1_/eV	0.017723	0.18833	0.1788
*A*_1_	51.217	22.996	73.251
*ω*_02_/eV	4.1655	3.3400	3.5362
Γ_2_/eV	0.18819	0.68309	0.35467
*A*_2_	30.770	57.540	40.007
